# Correlation Between Ambulatory Blood Pressure Monitoring and Target Organ Damage in Children

**DOI:** 10.3390/children13070955

**Published:** 2026-07-20

**Authors:** Musa Öztürk, Batuhan Bakırarar, Zeynep Birsin Özçakar, Nilgün Çakar, Beyza Doğanay, Ercan Tutar, Fatoş Yalçınkaya

**Affiliations:** 1 Department of Pediatrics, Ankara University Faculty of Medicine, Ankara 06100, Türkiye; 2 Department of Biostatistics, Ankara University Faculty of Medicine, Ankara 06100, Türkiye; 3 Division of Pediatric Nephrology, Ankara University Faculty of Medicine, Ankara 06100, Türkiye; 4 Division of Pediatric Cardiology, Ankara University Faculty of Medicine, Ankara 06100, Türkiye

**Keywords:** ambulatory blood pressure monitoring, childhood hypertension, target organ damage, left ventricular hypertrophy, machine learning

## Abstract

**Highlights:**

**What are the main findings?**

- Elevated 24-h ambulatory systolic blood pressure was independently correlated with left ventricular hypertrophy; however, exploratory machine-learning models using ABPM parameters to assess comprehensive target organ damage risk showed limited discrimination.
- No independent association was observed between ABPM metrics and hypertensive retinopathy.

**What are the implications of the main findings?**

- ABPM-derived systolic parameters may support cardiovascular risk stratification and help identify children who need closer evaluation for target-organ damage.
- The proposed lower percentile thresholds and ABPM-based prediction models require prospective external validation before clinical implementation.

**Abstract:**

Background: Ambulatory blood pressure monitoring (ABPM) offers a comprehensive assessment of mean blood pressure (BP), circadian patterns, and out-of-office hypertension phenotypes. We evaluated the association of ABPM parameters with left ventricular hypertrophy (LVH) and hypertensive retinopathy (HRP) in children with hypertension. Methods: This retrospective analysis included 269 children who underwent ABPM at a tertiary care facility. Multivariable logistic regression was used to evaluate associations between BP Z scores and target organ damage (TOD), adjusting for age, sex, and body mass index standard deviation score. Machine learning techniques (logistic regression, random forest, and multilayer perceptron) were also evaluated using 10-fold cross-validation across exploratory combinations of the 90th or 95th percentiles and BP-load thresholds of 25% or 50%. Results: LVH was detected in 45/247 patients (18%) and HRP was observed in 22/240 patients (9%). The 24 h systolic BP Z score was independently associated with LVH (adjusted OR 1.44; 95% CI 1.16–1.80; *p* = 0.001). Cohort-derived systolic Z-score thresholds for LVH were 1.31 for 24 h systolic BP and 1.47 for nighttime systolic BP, corresponding to approximately the 90.6th and 93rd percentiles, respectively. No variable evaluated was independently associated with retinopathy. Logistic regression using the 90th percentile/25% load dataset showed the highest performance among the evaluated classifiers, but discrimination was limited (ROC area 0.603; MCC 0.041). Conclusions: Higher ambulatory systolic BP was independently associated with LVH but not with clinically detected hypertensive retinopathy. ABPM values at or above the 90th percentile and a systolic load of ≥25% may help identify children at increased risk of TOD, although these thresholds remain exploratory and require prospective external validation.

## 1. Introduction

Childhood hypertension is a chronic disease that can occur in all age groups, and its frequency increases with obesity [1]. Hypertension in children is generally asymptomatic and is identified by elevated blood pressure measurements obtained during routine outpatient visits. Elevated blood pressure can have secondary effects on several organs and systems, including the heart, kidneys, and eyes, due to the increased pressure load. It is crucial to prioritize follow-up and treatment, given the longer lifespan of hypertension in pediatric patients.

Ambulatory blood pressure measurement (ABPM) is commonly used in the management of children who exhibit hypertension during office blood pressure readings [2,3]. Normative values by age, height, and gender are used to analyze ambulatory blood pressure data [4]. Blood pressure parameters that are higher than usual can predict the degree of hypertension and secondary organ damage [5,6]. The management of childhood hypertension is based on evaluating average ambulatory blood pressures and assessing the development of secondary organ damage in hypertensive individuals. Our study aimed to examine the relationship between ABPM and the occurrence of left ventricular hypertrophy and retinopathy, both outcomes of secondary organ damage in patients with hypertension.

## 2. Materials and Methods

### 2.1. Study Design and Patient Selection

This study was conducted at Ankara University Faculty of Medicine, specifically in the Department of Pediatric Nephrology, among individuals diagnosed with hypertension. A total of 312 pediatric patients with hypertension who attended an outpatient clinic over a 6-month period were included in the study. According to the American Academy of Pediatrics, hypertension is defined as a blood pressure at or above the 95th percentile for children under 13 years of age and at or above 130/80 mmHg for those aged 13 and older, measured across three separate visits using appropriate equipment and positioning [2]. Every patient had a single pre-treatment assessment. Approval was granted from the Ankara University Faculty of Medicine Clinical Research Ethics Committee (i3-90-19).

A retrospective review of patients’ files and electronic records was conducted. The demographic details, including age, gender, height, and body weight, as well as the cause of admission to the hospital, information gathered from ambulatory blood pressure monitoring, the presence of left ventricular hypertrophy, and retinopathy were collected.

### 2.2. Ambulatory Blood Pressure Monitoring

Patients who were at least 5 years old and had a height of at least 120 cm underwent ABPM. ABPM was recorded using the Oscar 2 device (Model 222; SunTech Medical, Morrisville, NC, USA), and data were interpreted using Spacelabs analysis software (v90217A; Spacelabs Healthcare, Snoqualmie, WA, USA). The device, attached to the child’s non-dominant arm with an appropriate cuff, automatically collected measurements at 15–20 min intervals during the day and 30 min intervals at night. To ensure the validity of ambulatory blood pressure monitoring, a minimum of 40 readings should be obtained, with at least 8 recorded during nighttime hours. Only recordings with at least 70% valid measurements and sufficient nighttime readings were analyzed, with nocturnal periods defined based on sleep diaries. Using the computer software, measurements equal to or above the 95th percentile by age and gender were categorized as hypertensive and marked for “systolic and diastolic load” calculation [2].

The ratios of hypertensive readings to all measures were calculated separately for day and night, and the result is referred to as “load” [7]. A 25% and above “load” was considered hypertensive. In individuals, average systolic blood pressure measurements tend to be 10–20% lower during nighttime hours compared to daytime hours. We identified “dipping” by calculating and quantifying the ratio of the day-to-night mean systolic blood pressure difference. A reduction of less than 10% of this “dipping” ratio was referred to as a “non-dipper” [8]. The blood pressure data obtained was processed via the LMS approach as described in the study of Wühl et al. [4]. We converted each patient’s blood pressure into an exact standard deviation score (SDS) using the relevant median (M), coefficient of variation (S), and measure of skewness (L) [9]. Z scores were calculated for the age and height datasets independently, and all statistical analyses were conducted for both groups. The Z scores obtained were converted into percentile values on the one-sided normal distribution graph in reverse.

### 2.3. Target Organ Damage (TOD)

Two-dimensional and M-mode echocardiographic examinations were performed using a Vivid 7 Pro ultrasound system (GE Vingmed Ultrasound AS, Horten, Norway). The tests were conducted by an experienced pediatric cardiologist who was unaware of the blood pressure readings, according to the guidelines provided by the American Society of Echocardiography. We computed the left ventricular mass using the Devereux method [10]. Left ventricular hypertrophy was defined using the age-, sex-, and height-specific reference nomograms previously described by Khoury et al. [11].

Hypertensive retinopathy (HRP) encompasses the retinal microvascular manifestations that arise from elevated systemic blood pressure. In the pediatric population, it serves as a vital indicator of TOD [12]. The retina offers a distinctive opportunity to observe systemic microcirculation non-invasively. In pediatric patients, hypertensive retinopathy is characterized by arteriolar narrowing, arteriovenous nicking, cotton-wool spots, hemorrhages, or optic disc edema associated with hypertension [13]. A single specialized ophthalmologist assessed retinopathy using the Keith–Wagener–Barker classification method by dilated fundoscopic examination [14].

### 2.4. Analysis of Data

Continuous data were summarized by mean ± standard deviation (SD) and median (with minimum and maximum values). Frequency and percentage were used for categorical data.

The Shapiro–Wilk test was used to evaluate the conformity of continuous data to a normal distribution. Independent Sample T-test or Mann–Whitney U was used to compare the two groups regarding numerical data. The receiver operating characteristic (ROC) curve was used to assess the diagnostic accuracy of the diagnostic test. After specifying an optimal cut-off value by Youden’s Index, specificity and sensitivity were calculated. The Chi-Square and Fisher’s exact tests were employed to compare nominal variables in cross-tables. Statistical analyses were performed using IBM SPSS Statistics 23 software (IBM Corp. in Armonk, NY, USA).

Multivariable logistic regression analysis was performed to evaluate independent associations between ambulatory systolic blood pressure parameters and target organ damage outcomes (left ventricular hypertrophy and retinopathy). Age, sex, BMI SDS, and 24 h systolic blood pressure Z scores were entered simultaneously into the models. Odds ratios (ORs) with 95% confidence intervals (CIs) were calculated, and statistical significance was set at *p* < 0.05. Model calibration was assessed using the Hosmer–Lemeshow goodness-of-fit test, which compares observed and predicted event frequencies across deciles of predicted risk.

The three classification algorithms were selected to compare an interpretable linear model, logistic regression, with a tree-based ensemble method, random forest, and a neural-network approach, multilayer perceptron, which can capture nonlinear associations and interactions. Classification methods of multilayer perceptron, logistic regression and random forest were used in the WEKA program. The data set was evaluated using the 10-fold cross-validation test option. Accuracy, F-Measure, Matthew’s correlation coefficient (MCC), precision–recall curve (PRC Area) and ROC area were used as data mining performance criteria. Descriptive statistics were calculated using R statistical language (version 4.1.0), whereas WEKA 3.8 version was used for classification analysis. Accuracy is the correct classification percentage of the model in the prediction of left ventricular hypertrophy and/or hypertensive retinopathy. F-Measure is the model’s accuracy, which is calculated as a harmonic mean with positive predictive value (PPV) and sensitivity. F-measure ranges in 0 to 1, where 1 means perfect PPV and sensitivity and 0 means either PPV or sensitivity is zero. ROC area is the degree or measure of separability of patients regarding left ventricular hypertrophy and/or hypertensive retinopathy. Area ranges between 0 and 1. A model whose predictions are 100% wrong has an ROC area of 0; one whose predictions are 100% correct has an ROC area of 1.0. Precision–recall curves plot the PPV against the sensitivity. A model whose positive predictions (i.e., patients having left ventricular hypertrophy and/or hypertensive retinopathy are 100% wrong has a PRC area of 0; one whose positive predictions are 100% correct has a PRC area of 1.0).

## 3. Results

A total of 312 hypertensive patients, with a median age of 13 years (range, 2–18 years) and a median follow-up of 16 months (range, 6–195 months) participated in the study. In total, 181 (58%) patients were male; 150 (48%) were obese and 58 (19%) were overweight (Table 1).

ABPM assessments were carried out on 269 pediatric patients. ABPM data indicate that 187 (69.5%) children had average blood pressure exceeding the 95th percentile in their 24 h, daytime, or nighttime measurements. When 25% or more were described as “load”, daytime systolic load was detected in 180 (67%) patients. A nighttime pressure drop of less than 10% was found in 133 (49%) patients. Including all ABPM parameters, as well as the concepts of “non-dipper” and “load”, 243 (90.3%) patients have hypertensive ABPM values (Supplementary Figure S1).

Left ventricular hypertrophy was observed in 45 out of 247 patients (18%) who underwent cardiac evaluation, while hypertensive retinopathy was detected in 22 of 240 patients (9%) who had fundoscopic exams.

Data from 206 patients who underwent both cardiac examinations and ABPM were analyzed. In the cardiological assessment of 206 children who underwent ABPM evaluation, LVH was present in 37 (18%) patients. There is a significant correlation between daytime and nighttime systolic “load” increase and cardiac hypertrophy (*p* values 0.033–0.047). Daytime systolic load threshold value was calculated as 53% (sensitivity 73%, specificity 53%) and nighttime systolic load threshold value was calculated as 31% (sensitivity 81%, specificity 40%) for left ventricular hypertrophy.

Z scores derived from ABPM data were evaluated in relation to the presence of left ventricular hypertrophy and retinopathy. The mean systolic Z scores, calculated from height and age, correlated with LVH (Table 2). Z scores of ABPM averages and left ventricular hypertrophy were found to be continuous variables with normal distribution in systolic 24 h, daytime and nighttime measurements. Considering the data presented a normal distribution, the subsequent threshold values were computed: the 24 h mean systolic Z score threshold value was calculated as 1.31 (90.6% percentile equivalent) and the night systolic Z score threshold value was calculated as 1.47 (93% percentile equivalent) according to height for LVH presence. Diastolic measurements do not conform to a normal distribution with left ventricular hypertrophy. Fundoscopy evaluation of 202 patients who underwent ABPM assessment revealed retinopathy in 18 (9%) patients. No statistically significant correlation was found between the presence of retinopathy and any of the ABPM parameters.

Multivariate logistic regression was used to identify independent predictors of left ventricular hypertrophy (LVH) and retinopathy (Table 3). In the LVH model, after adjustment for age, sex, and BMI SDS, the 24 h systolic blood pressure Z score emerged as an independent predictor of LVH (OR: 1.44, 95% CI: 1.16–1.80, *p* = 0.001). Neither age (OR: 0.94, 95% CI: 0.84–1.05, *p* = 0.265), male sex (OR: 1.93, 95% CI: 0.87–4.25, *p* = 0.104), nor BMI SDS (OR: 0.85, 95% CI: 0.62–1.16, *p* = 0.293) was independently associated with LVH. The Hosmer–Lemeshow goodness-of-fit test indicated adequate calibration of the model. ROC analysis demonstrated that the model showed acceptable discriminative performance for identifying LVH.

In the retinopathy model, none of the examined variables were independently associated with retinopathy. Age (OR: 0.96, 95% CI: 0.83–1.12, *p* = 0.622), male sex (OR: 1.06, 95% CI: 0.39–2.87, *p* = 0.907), BMI SDS (OR: 0.72, 95% CI: 0.50–1.06, *p* = 0.094), and 24 h systolic Z score (OR: 1.25, 95% CI: 0.94–1.67, *p* = 0.125) did not reach statistical significance. The Hosmer–Lemeshow test indicated inadequate calibration for this model. ROC analysis indicated limited discriminative ability of the retinopathy model. Overall, increased 24 h systolic blood pressure burden was independently associated with LVH but not with retinopathy in this cohort.

**Figure 1:**
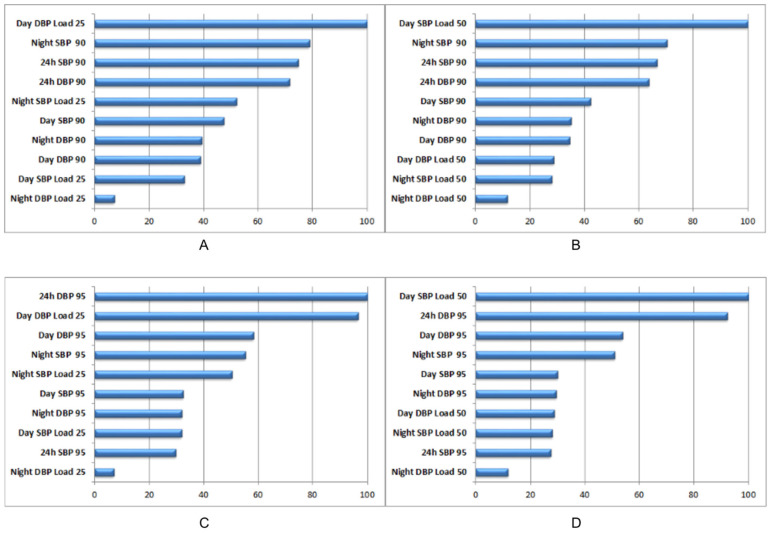
Relative variable-importance plots for the exploratory classification models.

Patients were grouped into two categories based on the presence of hypertensive left ventricular hypertrophy and/or retinopathy, a target organ damage (TOD) indicator, for the data-mining study. Hypertension was defined as being present at 24 h/day/night systolic/diastolic measurements above the 90th and 95th percentiles and was compared in terms of TOD. Day/night systolic/diastolic “load” beyond 25%/50% was also compared in terms of TOD (Supplementary Table S1). At further investigation, four different data sets were created on these groups, with ABPM parameter positivity, mean blood pressures at the 90th and 95th percentiles, and “load” at 25% and 50%. Four groups were defined as ≥90p + 25% load (group A), ≥90p + 50% load (group B), ≥95p + 25% load (group C), and ≥95p + 50% load (group D). The groups received a performance analysis that applied data mining methods to assess the presence of TOD (Supplementary Table S2). When all data sets are examined, the model with the highest observed performance among the evaluated algorithms is logistic regression. When comparing the data sets, the one with the best performance is found to be group A (≥90p + 25% load). The variable importance graphs for the 90–25 data set are given in Figure 1A, for the 90–50 data set in Figure 1B, for the 95–25 data set in Figure 1C, and for the 95–50 data set in Figure 1D independently (Figure 1).

A key limitation of the present study is the absence of an external validation cohort. Although 10-fold cross-validation provides an internal estimate of model performance, it does not establish external validity. Consequently, neither the suggested percentile criteria nor the classification models can be expected to extrapolate to groups with varying demographic characteristics, hypertension etiologies, disease severity, or ABPM protocols.

Figure 1 shows the mean systolic blood pressure (SBP) and diastolic blood pressure (DBP) and load values of the study groups, as well as the relative importance of the variables within them (independent of each other) based on the presence of target organ damage. The groups are defined as follows: Group A: ≥90p + 25% load, Group B: ≥90p + 50% load, Group C: ≥95p + 25% load, and Group D: ≥95p + 50% load. Upon comparing the data sets, Group A demonstrates the best performance. The graphs indicate that all anticipated variables are significant predictors.

## 4. Discussion

Ambulatory blood pressure monitoring (ABPM) is the preferred method for assessing pediatric hypertension because it identifies white-coat hypertension and masked hypertension and provides comprehensive circadian blood pressure profiles. Our investigation revealed a robust correlation between ABPM values and the presence of left ventricular hypertrophy (LVH), a marker of early cardiovascular target organ damage (TOD).

Our cohort comprised 312 children, with a median age of 13 years, and 58% of the study group were male. This aligns with demographic trends observed in extensive pediatric hypertension studies, including that of Sorof et al., which indicated a male predominance and a greater incidence among adolescents [15,16]. Obesity was detected in 48% of our patients, a finding similar to other studies that associate elevated body mass index with increased blood pressure in children. The Bogalusa Heart project and recent findings from the SEARCH project validate that obesity is an important predictor of hypertension in teenagers [17]. Nighttime ABPM measurements in pediatric patients require careful evaluation. The suitability of the measurements, circadian rhythm, and the timing of nocturnal assessments are evaluated in conjunction with the sleep diary. In studies similar to ours, the incidence of nocturnal fall loss ranges from 50% to 60% [18,19,20].

The outcome analyses were conducted on partially overlapping complete-case subgroups because ABPM, echocardiogram, and fundoscopic examinations were not administered to all enrolled patients. This may have introduced selection and verification bias, limiting the direct generalizability of the observed prevalence and correlations to the full cohort.

The prevalence of LVH was 18% among patients who underwent echocardiography. This prevalence correlates with the 15–25% range observed in ABPM-based groups by Foster et al. and Mitsnefes et al., depending on the definition and indexing method used [21,22,23]. Our study specifically demonstrated that the daytime systolic load of ≥53% and the nocturnal systolic load of ≥31% are significant predictors of left ventricular hypertrophy (LVH). This study treated load as a historical and exploratory exposure variable to facilitate comparison with previous pediatric studies, rather than arguing for its incorporation into the current classification system [3]. These thresholds are comparable to those reported by Sorof et al., who found that the systolic load >50% was significantly associated with increased left ventricular mass index in children [24]. Consistent with our research, Wu et al. established a correlation between elevated systolic load and LVH in children. Although a comparable correlation is shown in adult patients, numerous researchers prioritize loading rates above 50% [25,26]. Considering that the cut-off values for nighttime systolic load determined in our study were 31% (sensitivity 81%, specificity 40%), a threshold of 25% for systolic load may be thought to be needed to avert preclinical cardiac implications. Nevertheless, the available evidence indicates that Hamdani et al.’s research shows no advantage in using “load” (combined mean arterial pressure) to predict LVH detection [27]. The new data have prompted a change in the recommendations to simplify the interpretation of ABPM by removing pressure load [3]. In all the studies conducted, the “load” assessment was based on blood pressure readings exceeding the 95th percentile on the measuring device. In the next part of our study, the “load” might appear more significant when using the 90th percentile to project TOD. This could be seen as a limitation of our research, and future work could explore this further.

The study we performed encompassed exact percentiles of blood pressure readings and Z scores obtained from the LMS method formulated by Wühl et al. [4]. Given that our ABPM data demonstrated statistical significance in relation to the development of LVH and the Z score adhered to a normal distribution, the 24 h systolic 90.6 percentile and the nighttime systolic 93 percentile were calculated as the thresholds for LVH development. Numerous studies have shown that increased 24 h mean systolic and diastolic blood pressure readings, especially those exceeding the 95th percentile for age, sex, and height, are significantly correlated with increased left ventricular mass (LVM) and the development of left ventricular hypertrophy (LVH) in children and adolescents [28,29,30]. A recent longitudinal “The SHIP-AHOY study” cohort research revealed that children with ABPM systolic pressures above 95th percentile exhibited an elevated risk of developing left ventricular hypertrophy and other target organ damage over time [31]. Our results indicate that using the 90th percentile blood pressure threshold value may statistically aid in preventing the onset of LVH. Although diastolic readings did not show a significant correlation with LVH in our group, other studies have similarly indicated that systolic parameters are more predictive of cardiac hypertrophy in pediatric patients, perhaps because systolic parameters have a greater hemodynamic impact on left ventricular afterload [24,32,33]. This emphasizes the potential advantage of earlier intervention and closer follow-up in children presenting blood pressure values within the “high-normal” spectrum.

The thresholds established in this cohort suggest that cardiovascular risk may be elevated below the normative 95th percentile, but they were developed and assessed within the same selected group. Consequently, they should be considered exploratory risk indicators rather than alternative diagnostic or therapeutic benchmarks.

Hypertensive retinopathy was noted in 9% of our group, which, while lower than cardiac involvement, aligns with findings showing that retinal vascular alterations in hypertensive children tend to be subtle and underdiagnosed in the absence of systematic retinal imaging [12,34,35]. The quantity of our cases was considered inadequate to establish a correlation between hypertension and the emergence of retinopathy. Given the limited patient population with retinopathy, the lack of an independent association should be viewed as equivocal rather than definitive proof of no correlation between ambulatory blood pressure and retinal microvascular damage. A 2009 Canadian study assessing 35 children with hypertension identified a 9% prevalence of retinopathy, in line with our findings [12]. A separate research evaluating 39 patients with stage 2 hypertension over a 30-year duration identified a prevalence of 18% [35]. The suboptimal performance of our limited retinopathy model is likely due to the small number of events, low statistical power, and the inability of traditional fundoscopy and a single ABPM recording to detect subtle or cumulative microvascular damage.

The clinical value of ABPM has significantly evolved. Nevertheless, considerable advancements in pediatric ABPM have occurred, but some critical questions persist unresolved. A significant debate arises to the definition of ambulatory hypertension in pediatric populations [36]. The results indicate that, although ABPM offers extensive data, the conventional ABPM hypertension threshold (95p) was considered inadequate for predicting target organ damage (TOD). The data mining analysis was performed on this patient group using four distinct data sets, establishing the hypertensive threshold at 90% to 95% and the loading threshold at 25% and 50% (Table S2 and Figure 1). The optimal performance in this specific statistical investigation was attained by logistic regression analysis in the 90–25p group. Figure 1A reveals that the main warning signs for TOD are daytime diastolic loading above 25% and an average systolic blood pressure of 90% or greater. Despite logistic regression yielding the highest performance among the assessed algorithms, its discriminatory power was limited, as evidenced by an ROC area of 0.603 and an MCC of 0.041. Moreover, the reported accuracy of 75.7% failed to exceed the percentage of patients without TOD in the examined dataset. Consequently, these models should be regarded as exploratory and do not currently offer substantial value for individual clinical decision-making.

The machine-learning analysis was designed as an exploratory proof of concept to determine whether the full spectrum of ABPM parameters could be integrated into an individualized estimate of target organ damage risk, rather than relying on a single BP measurement or threshold. An integrated model could provide risk categorization and help prioritize additional cardiac or ophthalmologic assessments. However, the modest ROC and MCC values observed in the present study indicate limited discriminative performance and no clear clinical advantage over conventional logistic regression. Therefore, these findings demonstrate the feasibility of developing an ABPM-based TOD risk-calculation model but do not establish a clinically applicable prediction tool. Before integrating such a risk calculator into standard clinical practice, larger prospective multicenter cohorts, model calibration, evaluation of clinical net benefit, and independent external validation are necessary. Future work for this study aims to expand the database and include more patients with target-organ involvement to create an advanced “Decision Support System.” This will enable precise calculation of TOD risk and effective management of hypertensive patients by using the full spectrum of ABPM parameters.

## 5. Conclusions

In conclusion, this study underscores the clinical utility of ABPM in the pediatric hypertensive population, particularly in identifying early TOD. Our data indicate that increased systolic blood pressure measures, including systolic load and Z-score-derived percentile thresholds, are strongly correlated with left ventricular hypertrophy. However, diastolic measures seem to be less predictive. The findings suggest that ABPM results at or above the 90th percentile may help identify children at elevated risk of TOD; nevertheless, these exploratory thresholds require prospective external validation before integration into clinical practice.

## Figures and Tables

**Table 1:** Demographic and anthropometric characteristics of study population (N = 312).

Variable		Cardiac Examination (n = 247)			Funduscopic Examination (n = 240)		
	LVH (n = 45)		Normal (n = 202)	RP (n = 22)		Normal (n = 218)	
Age, y	12.91 ± 3.45		12.54 ± 3.35	12.75 ± 2.7		12.59 ± 3.43	
Sex, male, %	32 (71%)		113 (56%)	12 (54%)		132 (60%)	
BMI SDS	1.24 ± 1.66		1.41 ± 0.97	1.43 ± 1.09		0.93 ± 1.42	
- Obese	28 (62%)		98 (49%)	11 (50%)		113 (52%)	
- Overweight	4 (9%)		42 (21%)	1 (5%)		43 (20%)	
Office Blood Pressure, systolic Z score, mmHg	2.87 ± 1.52		2.54 ± 1.54	2.61 ± 1.34		2.6 ± 1.56	
Office Blood Pressure, diastolic Z score, mmHg	2 ± 1.33		1.59 ± 1.34	1.74 ± 0.92		1.66 ± 1.39	
ABPM, Mean Systolic ≥ 95 persentile	29 (78%)		100 (59%)	14 (78%)		111 (60%)	
ABPM, Night dipping, %	8.45 ± 5.87		10.24 ± 5.73	10.51 ± 6.42		9.83 ± 5.73	
ABPM, Non-Dipper	83 (49%)		23 (62%)	7 (39%)		91 (%49)	

BMI: body mass index, SDS: standard deviation score, LVH: left ventricular hypertrophy, RP: retinopathy.

**Table 2:** Relationship between ambulatory blood pressure monitoring mean value Z scores (cut-off Z scores and percentages) and target organ damage.

ABPM Data		Cardiac Evolution			Fundoscopic Examination		
Z Score According to Height		LVH	Normal	*p* Value	Retinopathy	Normal	*p* Value
Systolic	**24 h**	**2.57 ± 2.21**	**1.7 ± 1.76**	**0.010**	2.13 ± 2.09	1.85 ± 1.79	0.580
		**^a^** **1.315 Z score**	**^b^** **90.6 *p***				
	**Day**	**2.18 ± 2.03**	**1.32 ± 1.64**	**0.020**	1.76 ± 1.99	1.47 ± 1.68	0.550
		**^a^** **2.89 Z score**					
	**Night**	**2.74 ± 2.12**	**1.68 ± 1.48**	**0.006**	2.45 (−3.01 + 5.05)	1.75 (−1.63 + 7.25)	0.293
		**^a^** **1.475 Z score.**	**^b^** **93 *p***				
Diastolic	24 h	0.86 ± 1.71	0.69 ± 1.34	0.560	1.22 (−3.05 + 4.04)	0.47 (−2.86 + 5.30)	0.370
	Day	0.26 ± 1.58	0.11 ± 1.21	0.580	0.56 (−2.77 + 3.18)	−0.04 (−3.4 + 3.58)	0.250
	**Night**	**1.47** **(−1.98 + 7.39)**	**0.91 (−2.67 + 6.51)**	**0.043**	1.23 (−1.98 + 7.39)	1.09 (−1.87 + 4.98)	0.620
Z score according to age							
Systolic	**24 h**	**2.3 ± 2.17**	**1.51 ± 1.71**	**0.040**	2.01 ± 2.19	1.63 ± 1.73	0.480
		**^a^** **1.395 Z score.**	**^b^** **91.8 *p***				
	**Day**	**1.97 ± 2.03**	**1.15 ± 1.56**	**0.020**	1.68 ± 2.02	1.28 ± 1.6	0.420
		**^a^** **1.605**	**^b^** **94.6 *p***				
	**Night**	**2.52 ± 2.16**	**1.59 ± 1.54**	**0.010**	2.16 (−3.68 + 4.98)	1.67 (−1.72 + 7.46)	0.220
		**^a^** **3.2 Z score**					
Diastolic	24 h	0.88 ± 1.7	0.65 ± 1.3	0.450	0.93 ± 1.62	0.67 ± 1.3	0.520
	Day	0.27 ± 1.54	0.10 ± 1.21	0.530	0.56 (−3.05 + 3.11)	−0.09 (−3.28 + 3.5)	0.240
	**Night**	**1.62 (−2 + 6.94)**	**1.01 (−2.55 + 7.52)**	**0.060**	1.49 ± 1.88	1.17 ± 1.28	0.490
**BP Loads**							
**Daytime systolic load**		**^c^** **53%**		**0.033**			
Daytime diastolic load		**^c^** 32.5%		0.189			
**Nighttime systolic load**		**^c^** **31.5%**		**0.047**			
Nighttime diastolic load		**^c^** 59.5%		0.949			

Mean ± standard deviation is used for normally distributed data; median along with the minimum and maximum values are provided otherwise. ^a^ Cutoff Z scores for the development of cardiac hypertrophy. ^b^ Percentile equivalent of relevant Z scores (the corresponding percentages for the sample Z scores are: 0 = 50%; 0.68 = 75%; 1.28 = 90%; 1.645 = 95%). ^c^ Cutoff percentages for the development of cardiac hypertrophy. Statistically significant values (*p* < 0.05) are shown in bold.

**Table 3:** Multivariate logistic regression results of target organ damage.

	**Model 1: Left Ventricular Hypertrophy ^1^**		
**Variable**	**OR**	**95% CI**	***p*** **-value**
Age	0.938	0.838–1.050	0.2646
Sex (Male)	1.927	0.874–4.253	0.1041
BMI SDS	0.845	0.618–1.156	0.2925
24 h systolic mean Z score	1.441	1.156–1.798	0.0012
	**Model 2: Retinopathy ^2^**		
**Variable**	**OR**	**95% CI**	***p*** **-value**
Age	0.963	0.829–1.119	0.6224
Sex (Male)	1.061	0.393–2.868	0.9070
BMI SDS	0.723	0.495–1.056	0.0937
24 h systolic mean Z score	1.253	0.939–1.671	0.1249

^1^ Hosmer–Lemeshow Test: χ^2^ = 5.673, *p* = 0.6838. ^2^ Hosmer–Lemeshow Test: χ^2^ = 16.509, *p* = 0.0356.

## Data Availability

The data presented in this study are available on request from the corresponding author. The data are not publicly available due to institutional restrictions.

## Supplementary Materials

The following supporting information can be downloaded at https://www.mdpi.com/article/10.3390/children13070955/s1, Figure S1: Clinical evaluation summary of patients in the study; Table S1: Descriptive statistics of variable sets in terms of hypertensive organ damage; Table S2: Performance measures of data mining methods.

## Abbreviations

The following abbreviations are used in this manuscript:

ABPM	Ambulatory Blood Pressure Monitoring
BMI	Body Mass Index
BP	Blood Pressure
DBP	Diastolic Blood Pressure
ECG	Electrocardiography
GE	General Electric
IBM	International Business Machines
LVH	Left Ventricular Hypertrophy
LVM	Left Ventricular Mass
LVMI	Left Ventricular Mass Index
MCC	Matthew’s Correlation Coefficient
MLP	Multilayer Perceptron
PRC	Precision–Recall Curve
PPV	Positive Predictive Value
ROC	Receiver Operating Characteristic
SDS	Standard Deviation Score
TOD	Target Organ Damage
WEKA	Waikato Environment for Knowledge Analysis
Z score	Standard Score (in relation to height/age percentiles)
