# Supplementary material for: Correlation Between Ambulatory Blood Pressure Monitoring and Target Organ Damage in Children
Source: Children (Basel). 2026 Jul 20;13(7):955. doi: 10.3390/children13070955 (PMC13406871; doi:10.3390/children13070955)

Table S1: Descriptive statistics of variable sets in terms of hypertensive organ damage

**Variables**		**Left ventricular hypertrophy and/or hypertensive retinopathy (Target organ damage)**					
		**No**		**Yes**		**Total**	
		**n**	**%**	**n**	**%**	**n**	**%**
**Day Systolic Load 25%**	No	44	27.8	8	16.7	52	25.2
	Yes	114	72.2	40	83.3	154	74.8
**Night Systolic Load 25%**	No	45	28.5	7	14.6	52	25.2
	Yes	113	71.5	41	85.4	154	74.8
**Day Diastolic Load 25 %**	No	102	64.6	20	41.7	122	59.2
	Yes	56	35.4	28	58.3	84	40.8
**Night Diastolic Load 25%**	No	68	43.0	20	41.7	88	42.7
	Yes	90	57.0	28	58.3	118	57.3
**Day Systolic Load 50%**	No	77	48.7	12	25.0	89	43.2
	Yes	81	51.3	36	75.0	117	56.8
**Night Systolic Load 50%**	No	83	52.5	19	39.6	102	49.5
	Yes	75	47.5	29	60.4	104	50.5
**Day Diastolic Load 50%**	No	132	83.5	35	72.9	167	81.1
	Yes	26	16.5	13	27.1	39	18.9
**Night Diastolic Load 50%**	No	105	66.5	28	58.3	133	64.6
	Yes	53	33.5	20	41.7	73	35.4
**24hr Systolic Mean 90 percentile**	No	66	41.8	11	22.9	77	37.4
	Yes	92	58.2	37	77.1	129	62.6
**Day Systolic Mean 90 percentile**	No	74	46.8	15	31.2	89	43.2
	Yes	84	53.2	33	68.8	117	56.8
**Night Systolic Mean 90 percentile**	No	63	39.9	10	20.8	73	35.4
	Yes	95	60.1	38	79.2	133	64.6
**24hr Diastolic Mean 90 percentile**	No	109	69.0	24	50.0	133	64.6
	Yes	49	31.0	24	50.0	73	35.4
**Day Diastolic Mean 90 percentile**	No	128	81.0	33	68.8	161	78.2
	Yes	30	19.0	15	31.2	45	21.8
**Night Diastolic Mean 90 percentile**	No	92	58.2	21	43.8	113	54.9
	Yes	66	41.8	27	56.2	93	45.1
**24hr Systolic Mean 95 percentile**	No	76	48.1	17	35.4	93	45.1
	Yes	82	51.9	31	64.6	113	54.9
**Day Systolic Mean 95 percentile**	No	87	55.1	20	41.7	107	51.9
	Yes	71	44.9	28	58.3	99	48.1
**Night Systolic Mean 95 percentile**	No	80	50.6	16	33.3	96	46.6
	Yes	78	49.4	32	66.7	110	53.4
**24hr Diastolic Mean 95 percentile**	No	123	77.8	27	56.2	150	72.8
	Yes	35	22.2	21	43.8	56	27.2
**Day Diastolic Mean 95 percentile**	No	142	89.9	37	77.	179	86.9
	Yes	16	10.1	11	22.9	27	13.1
**Night Diastolic Mean 95 percentile**	No	106	67.1	26	54.2	132	64.1
	Yes	52	32.9	22	45.8	74	35.9

Table S2: Performance Measures of Data Mining Methods

Methods	Accuracy	F-Measure	MCC	ROC Area	PRC Area
	**the 90-25 dataset- group A**				
**Multilayer Perceptron**	0.709	0.670	0.015	0.536	0.665
**Logistic Regression**	0.757	0.677	0.041	0.603	0.698
**Random Forest**	0.714	0.664	0.017	0.538	0.664
	**the 90-50 dataset - group B**				
**Multilayer Perceptron**	0.704	0.658	0.034	0.464	0.626
**Logistic Regression**	0.757	0.677	0.041	0.553	0.679
**Random Forest**	0.699	0.631	0.149	0.456	0.623
	**the 95-25 dataset- group C**				
**Multilayer Perceptron**	0.714	0.669	0.004	0.518	0.663
**Logistic Regression**	0.743	0.669	0.005	0.577	0.686
**Random Forest**	0.714	0.652	0.066	0.459	0.622
	**the 95-50 dataset - group D**				
**Multilayer Perceptron**	0.689	0.669	0.033	0.533	0.665
**Logistic Regression**	0.738	0.659	0.051	0.547	0.664
**Random Forest**	0.704	0.676	0.041	0.542	0.672

Figure S1: Clinical evaluation summary of patients in the study

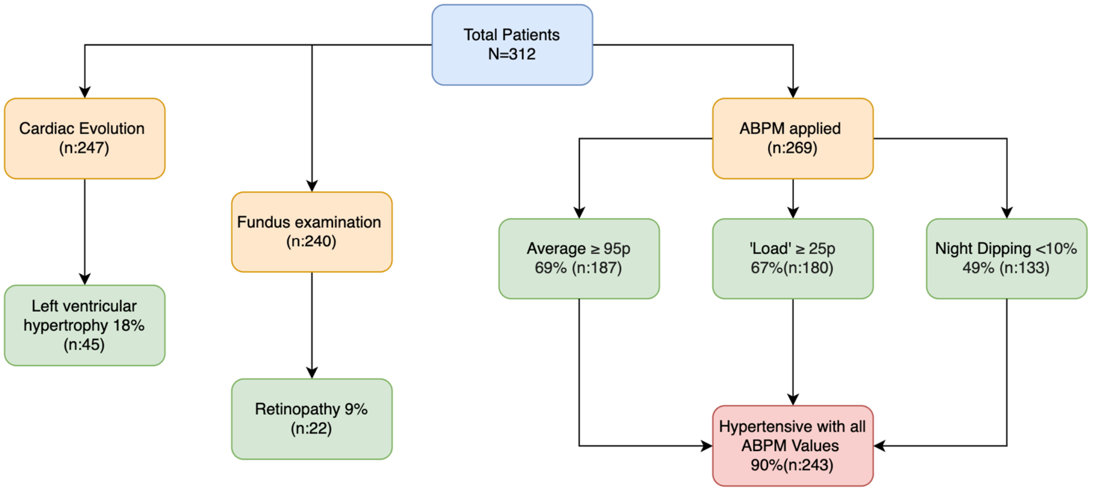

Supplement: Supplementary file 1 [file children-13-00955-s001.zip › children-4415254-supplementary.pdf]
